# Social Media Recruitment as a Potential Trigger for Vulnerability: Multistakeholder Interview Study

**DOI:** 10.2196/52448

**Published:** 2024-12-30

**Authors:** Nina Matthes, Theresa Willem, Alena Buyx, Bettina M Zimmermann

**Affiliations:** 1 Institute of History and Ethics in Medicine, TUM School of Medicine and Health, Technical University of Munich, Ismaningerstr. 22, Munich, 81675, Germany, 49 89 4140 4041; 2 Institute of Molecular Immunology, Klinikum Rechts der Isar, TUM School of Medicine and Health, Technical University of Munich, Munich, Germany; 3 Department of Science, Technology and Society (STS), School of Social Sciences and Technology, Technical University of Munich, Munich, Germany; 4 Institute of Philosophy, Multidisciplinary Center for Infectious Diseases, University of Bern, Bern, Switzerland

**Keywords:** vulnerability, social media, clinical study enrollment, clinical study recruitment, clinical trials, stigma, discrimination, injustice, recruitment, clinical study, hepatitis B, TherVacB, clinical research, attitudes, patient privacy, utilization

## Abstract

**Background:**

More clinical studies use social media to increase recruitment accrual. However, empirical analyses focusing on the ethical aspects pertinent when targeting patients with vulnerable characteristics are lacking.

**Objective:**

This study aims to explore expert and patient perspectives on vulnerability in the context of social media recruitment and seeks to explore how social media can reduce or amplify vulnerabilities.

**Methods:**

As part of an international consortium that tests a therapeutic vaccine against hepatitis B (TherVacB), we conducted 30 qualitative interviews with multidisciplinary experts in social media recruitment (from the fields of clinical research, public relations, psychology, ethics, philosophy, law, and social sciences) about the ethical, legal, and social challenges of social media recruitment. We triangulated the expert assessments with the perceptions of 6 patients with hepatitis B regarding social media usage and attitudes relative to their diagnosis.

**Results:**

Experts perceived social media recruitment as beneficial for reaching hard-to-reach populations and preserving patient privacy. Features that may aggravate existing vulnerabilities are the acontextual point of contact, potential breaches of user privacy, biased algorithms disproportionately affecting disadvantaged groups, and technological barriers such as insufficient digital literacy skills and restricted access to relevant technology. We also report several practical recommendations from experts to navigate these triggering effects of social media recruitment, including transparent communication, addressing algorithm bias, privacy education, and multichannel recruitment.

**Conclusions:**

Using social media for clinical study recruitment can mitigate and aggravate potential study participants’ vulnerabilities. Researchers should anticipate and address the outlined triggering effects within this study’s design and proactively define strategies to overcome them. We suggest practical recommendations to achieve this.

## Introduction

Vulnerability, defined as “an increased likelihood of being wronged or of incurring additional harm,” [1] has been a topic of significant interest in research ethics. Types of vulnerability include cognitive, juridic, deferential, medical, allocational, and infrastructural vulnerability [2]. The Declaration of Helsinki underscores that vulnerable populations require heightened protection due to their increased risk of harm, stating that research involving them is only justified when it directly addresses their health needs and cannot be conducted with nonvulnerable groups [1]. Yet, labeling whole population groups as vulnerable has been criticized as overly simplistic [3]. Accordingly, the revised Council for International Organizations of Medical Sciences guidelines from 2016 emphasize the importance of avoiding the exclusion of entire groups considered vulnerable from research participation under the guise of protecting their well-being, which has led to limited knowledge about diseases affecting these groups:

*In the past, groups considered vulnerable were excluded from participation in research because it was considered the most expedient way of protecting those groups (for example, children, women of reproductive age, and pregnant women). As a consequence of such exclusions, information about the diagnosis, prevention, and treatment of diseases that afflict such groups is limited. This has resulted in a serious injustice.… The need to redress these injustices by encouraging the participation of previously excluded groups in basic and applied biomedical research is widely recognized.*

[4]

To mitigate such effects, research ethicists suggested a more dynamic and context-dependent understanding of vulnerability. Luna [3] recognized that individuals may experience varying degrees of vulnerability and proposed that vulnerability is constituted by layers. Victor et al [5] emphasized the importance of identifying and addressing triggers that may exacerbate certain layers of vulnerability. Aggregating such layers of vulnerability might lead to cascading effects, a chain reaction of consequences that occur because of the activation of one vulnerability layer [5].

As such, these authors understand vulnerability as the sum of such layers (ie, personal characteristics or situational circumstances) that put a person at risk of being harmed or disadvantaged in specific contexts. An individual’s vulnerability is shaped by a combination of factors (eg, social, economic, and health-related) that can influence one another in ways that heighten or reduce overall vulnerability.

One potential factor that might aggravate—or mitigate—vulnerability in the research context is using social media as a recruitment channel for research studies. On the one hand, social media recruitment allows for effective reaching of populations that are challenging to reach through other recruitment channels since social media have targeted advertising features that enable researchers to tailor recruitment efforts to specific characteristics, interests, and behaviors. The accessibility of social media allows individuals to participate in research studies from anywhere with an internet connection, removing barriers related to geographic location, mobility limitations, or time constraints [6]. Thus, in specific contexts, social media recruitment may achieve higher enrollment rates and be more cost-effective than other recruitment methods [7]. On the other hand, however, social media can also involve considerable risks for specific target groups, including potential privacy violations, the risk of stigmatization, and challenges in ensuring proper informed consent [8]. Thus, to ensure ethical recruitment strategies, it is essential to identify how social media recruitment might trigger or mitigate vulnerabilities, especially in the context of clinical studies, and examine ways to address these triggering effects.

Previous studies investigating the ethical benefits and challenges regarding social media recruitment found that ethical benefits primarily include reaching a broader and more diverse pool of participants, and cost-effectiveness and direct engagement with potential participants. However, challenges such as ensuring data privacy, navigating regulatory compliance, and managing the quality of information dissemination require careful attention to maintain ethical standards [7-10]. Key recommendations from these studies stress the importance of ensuring recruitment strategies adhere to relevant ethical norms and comply with federal and state laws. Transparency is also crucial for building trust and upholding ethical standards throughout the recruitment process [10].

Despite these contributions, to our knowledge, no empirical studies specifically address vulnerability in the context of social media recruitment. For understanding the recruitment for clinical studies via social media as a “stimulus condition” [11] that potentially triggers or aggravates existing vulnerabilities, this paper aims to explore experts’ perceptions of vulnerability and those of patients with hepatitis B in the context of social media recruitment. We asked multidisciplinary experts and patients diagnosed with hepatitis B about their experiences and perceptions of recruiting vulnerable people through social media. We addressed the following research questions: (1) How can social media recruitment mitigate existing vulnerabilities in the context of clinical study recruitment? (2) What social media features can trigger and exacerbate vulnerabilities and why? (3) How do interviewees suggest navigating these triggering effects?

Chronic hepatitis B infection serves as a use case for vulnerability, underscoring the interplay between medical and psychosocial vulnerabilities experienced by affected individuals. Hepatitis B, a viral liver infection caused by the hepatitis B virus, leads to chronic liver disease with severe complications such as cirrhosis and hepatocellular carcinoma, presenting a significant public health challenge worldwide [12]. Individuals with hepatitis B encounter medical vulnerability due to their medically exigent state, compounded by the absence of a curative treatment. This renders them susceptible to exploitation, as they may enroll in clinical trials with inflated hopes of accessing potentially effective treatment. Their vulnerability is underscored by a persistent but often misguided hope for a cure, leading them to enter studies with unrealistic expectations of success [13]. Beyond medical vulnerabilities, individuals living with hepatitis B also face profound psychosocial challenges. Stigma and discrimination persist in many societies, driven by misconceptions about transmission routes and fear of contagion. This stigma manifests in various parts of life, including employment, health care settings, and social interactions, resulting in feelings of shame, isolation, and psychological distress [14].

## Methods

### Study Design

As part of the international research consortium “TherVacB - A Therapeutic Vaccine to Cure Hepatitis B,” we conducted semistructured interviews with multidisciplinary experts and patients with hepatitis B to explore the ethical, social, and regulatory issues of social media recruitment for clinical studies. In this paper, the analysis focused especially on the aspects of vulnerability. We followed the COREQ (Consolidated Criteria for Reporting Qualitative Research) checklist to report on the research team, study design, and data analysis [15].

### Ethical Considerations

#### Human Subject Ethics Review Approval

This study received approval from the Ethics Committee of the Technical University of Munich (431/20 S-KH).

#### Informed Consent

All participants were provided with an information sheet detailing the study’s purpose and scope. Written informed consent was obtained from each participant before the interviews.

#### Privacy and Confidentiality

Interview transcripts were pseudonymized by replacing identifiable information with placeholders.

#### Compensation Details

Participants did not receive any financial compensation for their participation in the study.

### Recruitment

Stakeholders with practical or theoretical experience in social media–based recruitment were eligible to participate. They were recruited via snowballing, convenient sampling through the TherVacB network, and screening the corresponding authors of relevant scientific publications in the field. The stepwise recruitment process was guided by considerations of theoretical saturation [16] and was stopped when additional interviews were not expected to reveal any relevant new findings based on the ongoing data analysis.

Moreover, we included patients with hepatitis B as an additional stakeholder group. Patients with hepatitis B aged 18 years and above with at least one social media account and English or German language skills were qualified to participate. They were recruited during regular hepatitis B-related check-ups at a German University Hospital. Due to the COVID-19 pandemic, patient recruitment was challenging. Therefore, no more than 6 patients with hepatitis B participated in this study. Consequently, theoretical saturation is limited for the patient population alone. We considered the insights from these interviews as an additional triangulation point to further substantiate our qualitative analysis.

### Data Collection

TW and BMZ held the semistructured, qualitative interviews in German or English via phone or videoconferencing. We used separate interview guidelines for experts and patients with hepatitis B. The expert interview guide focused on the ethical, legal, social, and practical risks and benefits of social media recruitment. The patient interview guide included questions about their experiences with social media and their disease, drawing particular attention to the potential stigma and privacy issues as essential aspects of vulnerability in the context of hepatitis B and social media. Experts were also asked “The patients eligible for the TherVacB clinical trial often have particularly vulnerable characteristics (eg economic, social). What do you think is noteworthy to consider in this context?”

The interviews were conducted between August 2020 and September 2021. Each interview lasted between 25 and 60 minutes. Interviews were recorded and transcribed verbatim.

### Data Analysis

The research team (BMZ; TW; Nina Goldman, PhD; and NM) transcribed audio recordings verbatim. Interview transcripts were coded based on inductive thematic analysis [17,18], using Atlas.ti (version 9; Scientific Software Development GmbH) software. Interview transcripts were analyzed based on reflexive thematic analysis (see Multimedia Appendix 1 for the final list of codes). Based on the first 6 expert interviews, BMZ and TW developed a preliminary coding scheme, which they applied to these 6 interviews. Then, they refined and reviewed the coding scheme in subsequent interviews until they identified no additional relevant codes. Next, the research team summarized the contents of each code across all interviews in analytical memos and related them to each other, with existing literature and theory. Thereby, we found vulnerability to be a central aspect of the interviews that warrants specific empirical and conceptual attention. In multiple rounds of discussion, NM, BMZ, and TW discussed aspects pertinent to the data inductively and refined them iteratively. Illustrative quotes in German were translated to English by NM and double-checked by BMZ.

## Results

### Overview

We conducted qualitative interviews with 6 patients with hepatitis B and 30 multidisciplinary experts from clinical research, public relations, psychology, ethics, philosophy, law, and social sciences. The patients resided in Germany; the experts were from Australia, Canada, Germany, Spain, Switzerland, and the United States. The findings from the qualitative analysis of these interviews are structured as follows: first, we outline how experts perceived social media recruitment to help mitigate pertinent vulnerabilities (eg, being affected by a stigmatized condition or belonging to historically disadvantaged populations) in the context of clinical study recruitment. Second, we present four features of social media that may trigger vulnerabilities. Third, we present experts’ recommendations from the interviews on mitigating some of these triggering effects.

### How Social Media Recruitment Can Help Mitigate Vulnerabilities

#### Reaching Hard-to-Reach Populations

Many experts have emphasized the potential of social media recruitment to reach traditionally disadvantaged hard-to-reach populations for research effectively. The paternalistic approach, often excluding these groups under the guise of protection, has led to a lack of research findings tailored to their specific needs and circumstances. An ethicist cautioned against such paternalistic views of vulnerability, underscoring the importance of respecting individuals’ autonomy:

*I believe that…it is important that the truly crucial concept of vulnerability is not used in an overly paternalistic way. To [avoid] thinking that anyone vulnerable is generally not autonomous.*

[Ethicist 1, Switzerland]

Achieving inclusivity in research necessitates shifting from paternalistic protectionism toward empowerment and collaboration with vulnerable populations. Meaningful inclusion entails valuing the autonomy of vulnerable individuals and actively involving them as partners in the research process while safeguarding their rights. Social media recruitment emerges as a valuable tool in this context, enabling researchers to reach these hard-to-reach populations more effectively, thereby promoting inclusivity and equity in research participation. Another ethicist further stressed the tension between protecting and recruiting patients with vulnerable characteristics:

*And maybe at some point, you can say, you know, you shouldn’t even be using social media, at least maybe in certain ways to recruit those really kinds of stigmatized groups. Again, I think we have to keep the benefits in mind here due to our earlier discussion because some of these folks are going to be/ Maybe this is the only way you’re going to reach them, right? Well, it’s sort of/It’s going to be the best way for you to reach them, so I guess I’m committed to kind of finding ways to do it sort of safely.*

[Ethicist 3, United States]

Consequently, several expert participants, especially ethicists, perceived social media as a helpful tool to reach underserved and hard-to-reach populations for research studies [19].

#### Preserving Patient Privacy

Despite some large social media platforms’ reputation for having limited privacy standards [20], social media recruitment may, conversely, also preserve patient privacy. By using social media platforms, researchers can reach individuals experiencing stigmatized conditions or traits, such as hepatitis B, outside a personal, clinical setting. Social media users with stigmatized conditions or traits who view social media recruitment advertisements may, for instance, be more likely to participate in survey research that does not require direct contact with the research team. They may prefer online recruitment settings that offer increased discretion and anonymity.

*In our case, and I’m thinking about the HIV study I’m involved in, we are trying very hard to reach people who may not be in healthcare. When we talk about putting flyers up around the hospital or an outpatient clinic we are already excluding people that don’t have access to regular care. We may not want to show up at a venue known to be frequented for privacy purposes, so if someone is going through [inaudible] or dating website [inaudible] in a way that can protect their privacy while reaching them whenever they get a chance to see an ad.*

[Ethicist 2, United States]

As we will show below, however, the predominant view among interviewees was that social media recruitment infringed on user privacy. This indicates the importance of assessing recruitment strategies in a context-sensitive manner, particularly regarding data privacy. The context relevance of who exactly the target population is may determine the risks and benefits related to this privacy aspect.

### Social Media Recruitment as a Trigger for Vulnerabilities

#### Overview

This section is structured along the features of social media that can trigger and exacerbate (existing) vulnerabilities in the context of social media recruitment. The relative importance of vulnerabilities depends on this study’s design and the target group of the social media recruitment strategy. Interviewees gave a range of examples of characteristics they considered making people vulnerable, including having sexually transmitted or infectious diseases (eg, hepatitis B), being affected by severe or untreatable conditions (eg, cancer), holding stigmatized traits (eg, sexual orientation or psychological conditions), or identifying with historically disadvantaged populations (eg, immigrants or people of color). If targeted for study recruitment via social media, these individuals may face various harms, including social exclusion, discrimination, and limited access to opportunities. For instance, those with sexually transmitted or infectious diseases may experience social exclusion and stigma. At the same time, individuals with severe illnesses may encounter financial burdens and emotional distress.

Similarly, individuals with stigmatized traits may confront prejudice, discrimination, and barriers to employment. Moreover, historically disadvantaged groups may encounter systemic inequalities, social injustices, and unequal access to employment and health care resources. While these risks of harm also exist beyond the context of clinical study participation, social media recruitment might trigger or exacerbate them.

#### Acontextual Point of Contact

When promoting studies via social media, individuals may encounter recruitment advertisements at unexpected moments. In the context of medical research, interviewees emphasized that learning about a clinical study outside a clinical setting might cause distress or exacerbate existing emotional challenges in participants’ lives, triggering or aggravating existing vulnerabilities:

*You don’t know the timing of your reach out. You know, it could be something very disturbing. It could be not just inappropriate timing,…, but just inappropriate in the context of a person’s life.… So, there is this contextual aspect of…recruiting people on social media, which can be hugely problematic.*

[Ethicist 1, United States]

Relatedly, a communication specialist with practical expertise in social media recruitment deemed social media inappropriate for studies investigating severe, uncurable diseases when social media content was usually about fun and happiness because this could be off-putting for those affected. He underscored the potential mismatch between the typically light-hearted nature of social media content and the serious nature of such health conditions:

*I believe that certain studies should simply not be promoted on social media.... We have recently received a request regarding a blood cancer study it is about the fact that the patient cannot be helped.... I consider it very unethical to advertise such things on social media platforms like Facebook, Instagram, and TikTok, at least not to offer such advertising.*

[Communication specialist 3, Germany]

Moreover, it was mentioned that the acontextual and impersonal communication on social media increased the risk of therapeutic misconceptions and false hopes regarding receiving an effective or curative treatment through the clinical study. One interviewed patient with hepatitis B supported this by stating:

*We are always told that there is never a chance for us to cure this disease,...and sometimes this thought overwhelms me. I want to search on social media to see what can be done in this regard.*

[Patient 2 with hepatitis B, Germany]

Thus, acontextual communication through social media might trigger both medical vulnerabilities that come with severe, untreatable, or otherwise burdensome conditions and cognitive vulnerabilities, such as insufficient health literacy.

#### Public Space

A second feature of social media is that at least some communication happens in public, where others can see and interact with the content. This feature can breach peoples’ need for privacy and unwantedly expose the medical information of social media users, constituting a risk of discrimination and people’s right to medical data privacy. A Canadian ethicist explained that patients might find it intrusive and irritating if they received targeted messages about their condition without actively seeking that information. When people publicly announce their illness, they may not expect that researchers looking for study participants with specific conditions could encounter their shared information. Thus, sensitivity is required in the initial engagement with potential study participants via social media, as receiving a targeted advertisement or private message based on this public disclosure may cause discomfort and feel like an intrusion into their privacy:

*And so, I think the form of initial engagement has to be sensitive to the fact that people might not expect that they’ve made that information public to this particular audience when they made a public announcement. And so, if you’re targeting a particular condition to sort of barge in and say, hey, so-and-so with condition X, that might be chilling to the person who’s receiving that message. He might feel like, how do you know this? And that could be off-putting and change their experience.*

[Ethicist, Canada]

Several patients with hepatitis B confirmed the importance of privacy. They expressed fear of being exposed to their diagnosis on social media.

*If I were to post something about my Hepatitis B to someone somewhere [on social media].... They would essentially have something in writing from me. And they could forward it or repost it at any time.*

[Patient 1 with hepatitis B, Germany]

Regarding stigmatized medical conditions, a communication expert raised concerns about hate speech and the potential for discriminatory comments on social media platforms, which can be off-putting for people, meaning that it could discourage them from participating in this study. The challenge is to create recruitment posts that attract potential participants while avoiding harmful reactions in the comments section.

*But how do you implement [social media recruitment ads] in a way that firstly appeals to the patients, and how do I also manage to avoid the hatred from healthy participants who have no understanding of what it means to have Parkinson’s? Because if a person with Parkinson’s reads something like this and is exposed to such advertisements, if they read those comments, what do you think will happen? They will not sign up for this study, and that’s problematic.*

[Communication specialist 3, Germany]

Thus, the feature of many social media platforms to operate (partly) in a public communication sphere triggers vulnerability for people with high privacy needs, such as people with stigmatized traits who would not want to be exposed. Such privacy infringements can lead to cascading effects, making people with stigmatized traits or severe diseases more vulnerable due to unsettling comments from other users or risks of discrimination.

#### Biased Social Media Algorithms

A third feature of social media that might trigger vulnerabilities in the context of historical discrimination is biased algorithms on social media. Several participants mentioned the issue of social media algorithms being potentially biased and discriminatory toward disadvantaged social groups. Consequently, using such algorithms for clinical study recruitment could trigger vulnerabilities for these groups. For example, one US-based ethicist working for an institutional review board stated:

*…we see a ton of social media, almost for every major clinical trial, from big industry sponsors like Novartis, and they almost always have some kind of social media outreach. I think there has been a growing desire to address potential inequities, so I know that Facebook has been criticized for, you know, when you run a clinical trial ad, who exactly is it going to reach? Is it going to reach people who are underserved and historically disadvantaged? There has been criticism of the algorithms they use to target people for clinical trial recruitment. And I think there is some desire amongst Facebook even to try and do better on that point. But I don’t know/ I guess I haven’t seen it slowing down.*

[Ethicist 3, United States]

While there is no right to participate in a clinical trial, the systematic neglect of research participants with certain traits might lead to lower health care standards. This occurs because clinical trials that lack diversity may produce results that are not generalizable to the entire population. For instance, if clinical trials primarily include participants from specific demographics, the findings may not accurately reflect how treatments affect other groups, such as those with different ages, genders, ethnic backgrounds, or health conditions. This can result in less effective treatments or unforeseen side effects in underrepresented groups, thereby lowering the overall quality and equity of health care. This issue is particularly relevant because social media is often perceived as a means to reach populations that are otherwise hard to engage in research, such as minority communities, rural populations, or those with limited access to traditional health care settings. However, this perception does not always hold in practice, as algorithmic biases may limit the effectiveness of social media recruitment [21]. As a result, specific populations may remain underrepresented in clinical research, perpetuating the cycle of inequitable health care standards.

#### Technological Tool

Finally, social media, being technological tools, cause effects that might trigger existing vulnerabilities. Several interviewed ethicists pointed out that users needed “a certain technical expertise and familiarity in dealing with such media” (ethicist 2, Switzerland). Thus, some patients may be interested in clinical studies but lack the digital skills to access this information via social media. As one expert pointed out, this can be a matter of justice if individuals with insufficient digital literacy skills are excluded from clinical studies solely advertised on social media:

*Especially with vulnerable groups, it is certainly an issue: Who doesn’t even have the opportunity or the chance to either see or respond appropriately? In that sense, it is indeed a matter of justice, but it’s probably not immediately apparent under the label of justice. It might be framed differently.*

[Clinical researcher 3, Germany]

In addition to insufficient literacy, restricted access to relevant technology can render those lacking this access as more vulnerable as they do not have the same access to information as others:

*You need the hardware for it, internet access or a stable network, and so on. Many things are required, and by that, I may inadvertently exclude certain people who don’t have access, who can’t afford it, or who may be hesitant to engage or participate in something like this. And just like that, we have a selection bias again. I think we need to be careful here. It can even take on a discriminatory character if I only focus on such individuals.*

[Ethicist 2, Switzerland]

Because social media are technological tools that require skills and hardware, their use can systematically exclude specific populations from clinical studies. This may lead to lower health care quality and aggravate medical vulnerabilities because drugs might work less efficiently. In the following section, we will present experts’ suggestions for mitigating these triggering effects of social media recruitment.

### Mitigating Triggering Effects of Social Media Recruitment

#### Overview

The interviewed experts pointed to several practical recommendations that help mitigate the aforementioned triggering effects of social media recruitment.

#### Communicating Transparently

First, several experts pointed to the importance of transparency when communicating with potential research participants on social media. One ethicist referred to people with vulnerable characteristics as requiring a “higher standard of transparency and consent” (ethicist 1, United States). Investigators should, therefore, always be transparent about who they are and what they are contacting the person for, as a clinical researcher from the United States pointed out. To further support patient autonomy, researchers should make sure that patients can ask questions before consenting to this study:

*…at least granting the opportunity to ask questions. What risks it would entail for me, for example? Even if it is formulated in writing, there might be instances where someone doesn’t understand it or so.… So, providing information that is as comprehensible as possible, I believe that’s what it is about.*

[Social scientist 1, Germany]

While transparency in research communication is a fundamental principle across all recruitment methods, it is particularly important in the context of social media recruitment, as users on social media platforms may receive numerous messages and requests. Transparency about the nature of the contact can help recipients differentiate legitimate research inquiries from spam or phishing attempts. This transparency helps establish trust and credibility with potential participants. By contrast, recruitment for clinical studies featured in newspapers or on the radio typically undergoes critical observation by the editorial teams beforehand. A clinical researcher from Germany also emphasized the need for personal interaction “to make sure that the patients understand that the information they are about to give you might contain personal health issues” (Clinical researcher 2, Germany).

#### Analyzing Algorithm Bias

To address the issue of biased algorithms, experts suggested researchers collaborate with ethicists and social media experts to review and assess the algorithms used for recruitment, identifying any potential biases and working toward mitigating them. Researchers should actively engage with Facebook and other social media platforms to understand how their algorithms work and to be aware of any changes or updates that may impact the fairness and inclusivity of their recruitment offers. Depending on the social media platform, this could be laborious, as this expert acknowledged:

*But Facebook changes its rules every second day, and you have to have someone whose job it is to monitor those.… It’s really, really hard from a privacy and confidentiality point of view because you’re not in control of what Facebook does.*

[Social scientist 2, Australia]

#### Protecting Privacy

One ethicist from the United States emphasized the importance of raising awareness about the potential misuse of data on social media. He suggested that instead of solely focusing on finding ways to protect privacy, it would be beneficial to educate people about the challenges of maintaining privacy online:

*I feel as though we need to be much more proactive about creating tools to protect all sorts of people in social media space from harm that may result from the misuse of their data. Rather than trying to figure out ways/ I think it would be healthier if we educated people that they’ve given up a ton of privacy, and maintaining privacy by keeping your information inaccessible is hard in these spaces and that we need to create an environment that creates consequences for the misuse of people’s information. So we shifted towards that sort of dynamic, maybe [inaudible] have a better understanding that but limited when you’re participating in a lot of these spaces.*

[Ethicist 4, United States]

A clinical researcher with practical experience in social media recruitment explained that his research team did not allow comments on their Facebook page to prevent patients from sharing information *“*about their diagnosis status or ask[Ing] questions about medication management” (clinical researcher 3, United States). To prevent patients from inadvertently disclosing personal health information, it is advisable not to specifically target individuals with a particular diagnosis in recruitment posts on social media. A US-based ethicist suggested making a disclaimer saying that users should not directly reply to study-related social media advertisements. Still, experts acknowledge the challenge of protecting patient privacy on social media because platforms may change their privacy policy anytime.

#### Using Multichannel Recruitment Approaches

Because not everybody has access to social media and might not see information regarding relevant clinical studies, one ethicist from the United States suggested that it may be necessary to use alternative offline strategies to reach populations with limited social media access:

*Social media...may not be available to everyone.... So we have to sort of keep that in mind. There is a place where you might want to drive up and hand out flyers outside of a soup kitchen, depending on who you’re trying to talk to.*

[Ethicist 2, United States]

Therefore, researchers should consider the possible drawbacks of relying solely on social media as a recruitment method and instead devise an approach that integrates various strategies (both online and offline) to promote inclusivity in clinical studies and increase the likelihood of reaching a more diverse and representative sample of participants. In addition, one expert suggested learning the target population’s behavior to “track down what data is available in terms of what websites they are on” (ethicist, Canada). While the other presented strategies involve specific actions to mitigate the vulnerability-triggering effects of social media recruitment, multichannel recruitment consists of integrating online and offline recruitment methods to enhance inclusivity.

## Discussion

### Principal Findings

In many clinical studies, social media offer one of several ways to recruit potential study participants. Most often, participants are recruited within the clinical setting, through snowballing techniques, or via more traditional forms of advertisement such as billboards, newspaper advertisements, and community networks. Gelinas et al [10] argued for a nonexceptional approach to evaluating the ethical implications of social media recruitment because the same research ethics principles (respect for persons, justice, and beneficence) apply. Yet, as we delve into this discourse, we recognize the need for a deeper examination. In this context, we propose to extend Gelinas and colleagues’ [10] recommendations. We offer the first empirical evidence of a more nuanced assessment of social media recruitment regarding participant vulnerability by showing features specific to social media that might mitigate or trigger vulnerabilities of potential study participants in the context of clinical study recruitment.

We will now discuss how the identified vulnerability triggers of social media recruitment are exclusive to social media. The vulnerabilities associated with social media recruitment are not mere extensions of issues seen in more traditional recruitment approaches. Instead, social media themselves shape vulnerabilities due to their unique characteristics. We argue that social media uniquely combines ethically relevant features of other recruitment channels and, thus, warrants special attention and ethical scrutiny. First, social media are unique because they operate in a semipublic sphere [22]: within this sphere, content can straddle the line between public and private, creating a complex web of accessibility. For the average user, determining who can access which specific activities and digital traces on social media is not trivial [23], particularly when considering the constantly changing terms of use imposed by social media platforms. Second, and this distinguishes social media recruitment from other online recruitment activities, is the deeply personal nature of these platforms. Social media platforms serve as more than just spaces for sharing information; they also collect vast amounts of data about individuals, including their preferences, behaviors, and interests.

Users recognize that the content they encounter on these platforms is tailor-made for them based on their previous interactions and the data collected about them. This personalization sets social media recruitment apart from traditional methods such as billboard advertisements, which are generally not personalized. This individual targeting might be off-putting for users because they may feel that their privacy is being invaded, which is not a concern with more traditional recruitment approaches. Finally, social media recruitment poses unique challenges related to privacy and algorithmic discrimination [20]. The power wielded by social media platforms in the digital realm is unprecedented, and their ability to shape the recruitment process is substantial. These platforms’ algorithmic decisions can inadvertently contribute to inequalities in health care and research participation [21]. Thus, when researchers use nondisclosed social media algorithms for recruitment, they may unintentionally exacerbate vulnerabilities for marginalized communities by targeting specific demographic groups and inadvertently excluding others, potentially exacerbating disparities in health care access and research participation. The use of such algorithms for clinical study recruitment has the potential to trigger vulnerabilities for marginalized groups. It is essential to consider the far-reaching impact of social media platforms and their algorithms on fairness in the recruitment process. As social media are used to recruit potential study participants, these risks of triggering or exacerbating vulnerabilities exist independent of actual research participation. Consequently, potential research participants could be harmed even without consenting to participate in a study, making it even more important to consider vulnerabilities in the context of social media recruitment carefully.

Particularly in Europe, privacy concerns and related legal uncertainty often cause researchers to refrain from using social media as a recruitment tool for clinical studies [9]. Yet, to not aggravate existing injustice, researchers and research ethics committees should not completely refrain from social media recruitment out of concern for vulnerability [24]. This should be especially considered in the context of historically underserved, hard-to-reach populations and people with stigmatized conditions, who may benefit from the platform’s accessibility and reach. Consequently, we conclude from our findings neither promoting nor declining positions on the use of social media for clinical study recruitment. Instead, we highlight the importance of a context-sensitive assessment of study designs regarding the role of social media in triggering vulnerabilities and report on concrete ways researchers could develop appropriate safeguards tailored to address the specific types of vulnerability involved [2]. In developing such appropriate safeguards, assessing the risks and benefits of using social media recruitment regarding vulnerability is essential.

### Limitations

The results of this study should be considered against the background of some methodological limitations. Due to limited access to patients during the COVID-19 pandemic, only 6 patients were recruited. While we found during the analysis that they represented a high variability in terms of age, digital literacy, and attitudes toward technology and social media, additional interviews might have revealed more nuanced patient views. Additionally, this study’s focus on patients with hepatitis B introduces another limitation. The vulnerability and ethical considerations related to social media recruitment can differ substantially across various diseases and patient populations, thus the findings may not be directly applicable to other medical conditions. Yet, this study provides relevant insights for future studies investigating vulnerability in the context of social media recruitment in the context of other disorders.

Another potential limitation is that some of the interviews were held via the phone. The absence of visual cues might have hurt the richness and quality of the empirical data compared to face-to-face interviews, particularly for patient interviews. Due to the COVID-19 pandemic, it was not possible to conduct these interviews face-to-face.

Finally, throughout the interview process, we did not provide a specific definition of social media to the participants. Instead, we asked them about their understanding of social media. As a result, the practical recommendations presented in this paper are expected to have a more universal scope rather than being tailored to specific social media platforms. Thus, the provided recommendations may depend on the particular social media platform being considered, the target population, the kind of study being conducted, and the envisaged study design [8].

### Conclusions

The use of social media for clinical study recruitment can mitigate but also trigger or aggravate existing vulnerabilities. To avoid the systematic neglect of certain groups in research studies, vulnerability should be anticipated in the study design, and ways to mitigate them should be defined upfront. To facilitate this, we have reported a range of practical recommendations to address vulnerability and presented a practical case to do this. As such, social media recruitment should be designed and reviewed in a way to mitigate effects that render people more vulnerable. Our expert participants proposed that studies targeting people with stigmatized conditions or historically disadvantaged populations should make sure that the recruitment design allows for transparent communication and protection of privacy. Expertise in analyzing potential algorithm bias and using multichannel recruitment strategies are other practical recommendations for certain target populations.

## Supplementary material

10.2196/52448 Multimedia Appendix 1 Final list of codes.

## Abbreviations

COREQ: Consolidated Criteria for Reporting Qualitative Research
