# Supplementary material for: Social Media Recruitment as a Potential Trigger for Vulnerability: Multistakeholder Interview Study
Source: JMIR Hum Factors. 2024 Dec 30;11:e52448. doi: 10.2196/52448 (PMC11702910; doi:10.2196/52448)
Supplement: Multimedia Appendix 1 [file humanfactors-v11-e52448-s001.docx]

## Multimedia Appendix 1

## Final List of Codes

| **Code groups** | **Codes** |
| --- | --- |
| Benefits of SMR | Benefits of SMR |
| Ethics | Ethics_Cause harm / pain / disturbances / distress |
|  | Ethics_Data protection /privacy issues |
|  | Ethics_Discrimination / stigma |
|  | Ethics_Generalizability, selection bias, fairness |
|  | Ethics_growing awareness of ethical issues |
|  | Ethics_Transparency |
|  | Ethics_Vulnerable populations |
| Good research ethics | Research ethics_conflicts of interest |
|  | Research ethics_quality assessment |
| Legal | Legal_Regulatory issues |
| Patients' perspectives | Experts about patients acceptance of SMR |
|  | Patients_acceptance of SMR |
|  | Patients_experiences with SMR |
|  | Patients_social media literacy |
|  | Patients_therapeutic misconceptions |
|  | Patients_usage habits |
| Practical implications | Other practical challenges |
|  | Potential solutions for practical SMR problems |
|  | Resources needed to conduct SMR |
|  | SMR Effectiveness |
|  | SMR professionalization |
|  | SMR strategy development |
| SMR Particularities | Justifications pro/contra SMR |
|  | Particularities_clinical trials vs other research |
|  | Particularities_Europe/America |
|  | Particularities_SMR compared to other recruitment methods |
